# Test Performance Characteristics of Dynamic Liver Enzyme Trends in the Prediction of Choledocholithiasis

**DOI:** 10.3390/jcm11154575

**Published:** 2022-08-05

**Authors:** Yang Lei, B. Cord Lethebe, Erin Wishart, Fateh Bazerbachi, B. Joseph Elmunzer, Nirav Thosani, James L. Buxbaum, Yen-I Chen, Sydney Bass, Martin J. Cole, Christian Turbide, Darren R. Brenner, Steven J. Heitman, Rachid Mohamed, Nauzer Forbes

**Affiliations:** 1 Department of Medicine, Cumming School of Medicine, University of Calgary, Calgary, AB T2N 4N1, Canada; 2 Clinical Research Unit, Cumming School of Medicine, University of Calgary, Calgary, AB T2N 4N1, Canada; 3 CentraCare, Interventional Endoscopy Program, St. Cloud Hospital, St. Cloud, MN 56303, USA; 4 Division of Gastroenterology and Hepatology, Medical University of South Carolina, Charleston, SC 29425, USA; 5 Center for Interventional Gastroenterology (iGUT), McGovern Medical School, UTHealth, Houston, TX 77030, USA; 6 Division of Gastrointestinal and Liver Diseases, Keck School of Medicine, University of Southern California, Los Angeles, CA 90033, USA; 7 Department of Medicine, McGill University, Montreal, QC H3A 1A1, Canada; 8 Department of Community Health Sciences, Cumming School of Medicine, University of Calgary, Calgary, AB T2N 1N4, Canada; 9 Department of Cancer Epidemiology and Prevention Research, Cancer Control Alberta, Alberta Health Services, Calgary, AB T2S 3C3, Canada; 10 Department of Oncology, Cumming School of Medicine, University of Calgary, Calgary, AB T2N 1N4, Canada

**Keywords:** choledocholithiasis, ERCP, EUS

## Abstract

(1) Background: Various methods to predict the presence or absence of choledocholithiasis (CDL) have been proposed. We aimed to assess the performance characteristics of dynamic liver enzyme trends in the prediction of CDL. (2) Methods: This was a single-center retrospective cohort study. All adult in-patients undergoing endoscopy for suspected CDL between 1 January 2012 and 7 October 2018 were identified, with patients with prior cholecystectomy, prior sphincterotomy, or indwelling biliary prostheses were excluded. Available laboratory parameters within 72 h preceding the procedure were recorded, allowing for the assessment of trends. Dynamic enzyme trends were defined as any increase or decrease by 30% and 50% within 72 h of the index procedure. (3) Results: A total of 878 patients were included. Mean age was 61.8 years, with 58.6% female. Increases in alkaline phosphatase (ALP) of at least 30% or 50% were both specific for the presence of CDL, with specificities of 82.7% (95% CI 69.7–91.8%) and 88.5% (95% CI 76.6–95.6%), respectively. Decreases in bilirubin or ALP of at least 50% were highly specific for the absence of CDL, with specificities of 91.7% (95% CI 85.7–95.8%) and 100.0% (97.2–100.0%), respectively. (4) Conclusions: Several liver enzyme trends appear to be specific for the absence or presence of stones; in particular, significant decreases in total bilirubin or ALP of at least 30–50% over the prior 72 h appear to be especially predictive of an absence of intraductal findings during endoscopy.

## 1. Introduction

Choledocholithiasis (CDL) is a common clinical entity, observed in up to 20% of patients with symptomatic cholelithiasis [1]. Untreated or missed, CDL can lead to episodes of abdominal pain, jaundice, pancreatitis, and ascending cholangitis [2]. Endoscopic retrograde cholangiopancreatography (ERCP) is established as the first-line procedure for definitive management of CDL [3]. While highly effective, ERCP is associated with several adverse events (AEs), including but not limited to post-ERCP pancreatitis, bleeding, cholangitis, cholecystitis, perforation, and cardiopulmonary or anaesthesia-related events [4]. Given these risks, ERCP should be used as a therapeutic modality, with safer less invasive preliminary testing employed to determine the likelihood of CDL before committing a patient to ERCP.

Endoscopic ultrasound (EUS) and magnetic resonance cholangiopancreatography (MRCP) are safe and highly sensitive for CDL and considered the non-invasive gold standards [5]. However, MRCP and especially EUS may not be readily available in all centers. In addition, patients with certain comorbidities (e.g., pacemaker-dependent arrhythmias) or those with higher risk medical profiles (e.g., significant cardiorespiratory disease) might not be suitable for magnetic resonance imaging or should avoid endoscopic procedures whenever possible. Moreover, these diagnostic modalities are costly. Hence, non-invasive bedside markers of risk stratification are appealing.

Guideline-based criteria support the clinical prediction of CDL informed by a constellation of clinical, biochemical, and radiographic findings [3,6,7]. Some of the most widely employed criteria are summarized in Table 1. Other approaches have included using the same variables to create a scoring system, and using the same variables as inputs in an artificial neural network [8,9]. Although clinical prediction tools perform well in cases where a high pre-test probability of CDL exists [10,11,12], the test performance characteristics of intermediate pre-test probability criteria are generally suboptimal, with reported sensitivity, specificity, and overall accuracy of less than 50% [11,13]. In such scenarios, the likelihood of benefit is only moderate, while the risk profile of ERCP remains comparatively high.

Further refinements in non-invasive diagnostic strategies are needed to minimize the number of unnecessary non-therapeutic ERCPs among intermediate pre-test probability CDL patients. While abnormal liver biochemistry can be useful in predicting the presence of CDL, the role of “real-time” dynamic changes, or trends in liver enzymes, requires additional study [14,15]. Conceptually, dynamic liver enzyme trends would appear to offer a biologically plausible method for evaluating patients at intermediate risk of CDL. Dynamic changes may help stratify patients who are more likely to have passed a stone while awaiting ERCP. Optimizing the evaluation and management of these patients could improve patient outcomes and reduce health care expenditure. Thus, we aimed to study the performance characteristics of dynamic liver enzyme trends in the prediction of CDL, in addition to assessing the test performance characteristics of the 2010 ASGE, 2019 ASGE, and the 2019 ESGE criteria in a tertiary center cohort.

## 2. Materials and Methods

### 2.1. Study Design and Setting

This was a single-center retrospective cohort study conducted at the Peter Lougheed Center, a tertiary referral center in Calgary, Alberta, Canada providing ERCP and EUS services. All procedures in the study were performed by one of six endoscopists, each having performed over 1000 of the respective procedures, or by trainees under their direct supervision. More than 60% of ERCPs were performed for indications of suspected CDL [16]. This study was approved by our institutional research ethics board (REB18-1053).

### 2.2. Patients, Variables, and Outcomes

All adult in-patients (age ≥ 18 years) who underwent ERCP or EUS for suspected CDL at our center between 1 January 2012 and 7 October 2018 were identified. Out-patients were excluded given the low likelihood of having serial liver enzymes measured leading up to procedures. In-patients from outside our health region (and thus with incomplete access to electronic medical records) were also excluded. Patients with prior cholecystectomy, prior sphincterotomy, or indwelling biliary prostheses, and those undergoing ERCP or EUS for any indications other than suspected CDL (including cholangitis), were also excluded at the time of the initial database search. Any unsuccessful EUS or ERCP procedure was also excluded after manual review of the report. 

The endoscopy reporting program endoPRO IQ (Pentax Medical, Montvale, NJ, USA) was searched for all ERCPs and EUS procedures performed within the study period that met the above eligibility criteria. Next, a review of each of the endoscopy reports was performed, in addition to a review of the patients’ in-patient medical records. Selective filters based on language and automated entry fields were initially used to code clinical data where possible, with additional review of non-classifiable cases. A quality assurance audit via full chart review was manually performed on 15% of study entries.

Patient baseline demographics (age and sex) were recorded. In addition, any available laboratory parameters within 72 h preceding the procedure were recorded, allowing for the assessment of trends. The timeframe of 72 h and less was chosen because we felt that the most proximate values to the time of the procedure would yield the most accurate assessment for the presence or absence of CDL at the time of procedure, along with our observation after preliminary data analysis of the demographics that the vast majority of our patients did not have in-patient biliary enzymes measured between seven and four days prior to the procedure. The measured parameters included total and direct bilirubin, alanine transaminase (ALT), aspartate transaminase (AST), alkaline phosphatase (ALP), gamma-glutamyltransferase (GGT), and lipase. The reported findings from relevant diagnostic imaging tests performed in the preceding 60 days were also captured, including abdominal ultrasound (US), computed tomography (CT), and magnetic resonance imaging (MRI). These findings included common bile duct (CBD) size and the presence or absence of any intraductal stones or sludge. We included sludge as it has similar clinical sequelae as stones. Intra-procedural findings were also reviewed, with a positive diagnosis of CDL being defined as the presence of one or more stone(s) or any descriptors including sludge, debris, or microlithiasis on EUS or ERCP, as confirmed by the procedure report.

### 2.3. Statistical Analyses

Dynamic enzyme trends were defined as any increase or decrease by 30% and 50% of the most divergent total bilirubin, ALT, or ALP values within 72 h of the index procedure. Any change within the 72 h, including within 48, 24, and 12 h, are captured with this timeframe. A wider range of within 72 h was used as same or next-day ERCP access is not always available at our institution. Previous dynamic enzyme trend studies have chosen absolute value differences [14,17], absolute direction of trend [18], and relative changes of 20% [19] and 30% [20]. We selected 30% and 50% changes, independent of absolute value in relation to the upper limit of the reference range, to replicate the criteria for previous studies (for the 30%) as well as analyse a scenario where there was more certainty about the magnitude of the trend to overcome any doubts about the range of laboratory measurement variation in patients with values within the normal reference range (50%). We created density plots of the percent change in total bilirubin, ALT, and ALP for patients both with and without stones or sludge.

Patients were assigned to a baseline (pre-procedural) risk of CDL according to both versions of the ASGE criteria and the ESGE criteria based on the above data, using the laboratory parameters in closest proximity leading up to their procedures. Test performance characteristics (sensitivity, specificity, positive predictive value (PPV), and negative predictive value (NPV)) of the 2010 and 2019 ASGE criteria and of the 2019 ESGE criteria were calculated, as were the performance characteristics of dynamic trends in each individual liver enzyme and combinations of enzymes. All analyses were performed using R version 3.6.0 (R Foundation for Statistical Computing, Vienna, Austria).

## 3. Results

**Figure 1:**
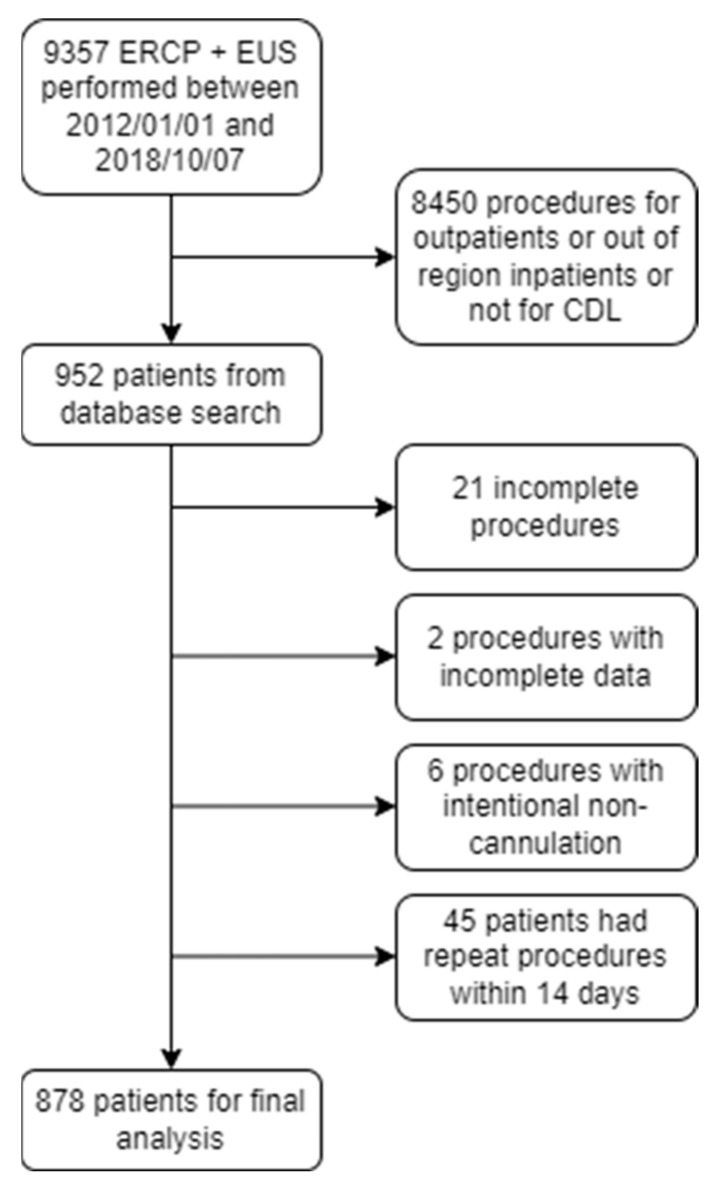
Patient selection flow diagram (CDL, choledocholithiasis; EUS, endoscopic ultrasound; ERCP, endoscopic retrograde cholangiopancreatography).

A total of 952 patients were included after applying exclusion criteria at the database search level. After manual review, an additional 74 procedures were excluded owing to a lack of procedural success, missing data, or previous procedure within 14 days, resulting in 878 patients being analyzed in the final cohort (Figure 1). Of these, total bilirubin at 72, 48, and 24 h preceding the procedure, and on the day of the procedure, were available for 195, 358, 565, and 538 patients, respectively. For ALP and ALT, these numbers were similar, at 191, 355, 557, and 536 patients and 195, 355, 555, and 535 patients, respectively, indicating that most liver enzymes are ordered as a grouped panel. The mean age of the cohort was 61.8 years, with 58.6% female and 41.4% male. In 622 cases, CDL was confirmed on ERCP or EUS (70.8% positive diagnosis rate). For context, 74 patients had a pre-procedure MRCP and 32 patients went on to have an intra-operative cholangiogram. A complete summary of baseline patient, procedure, biochemistry, and imaging parameters is provided in Table 2.

The test performance characteristics of *increases* in dynamic liver enzyme in the 72 h preceding the procedure are summarized in Table 3, where both a minimum 30% increase and minimum 50% increase in enzymes were considered. Increases in ALP of at least 30% or 50% were both specific for the presence of CDL, with specificities of 82.7% (95% CI 69.7–91.8%) and 88.5% (95% CI 76.6–95.6%), respectively. The combination of bilirubin and ALT both increasing by at least 50% was also specific, with a specificity of 84.3% (95% CI 71.4–93.0%). Dynamic enzyme increases had low-moderate PPVs for CDL, ranging from 57.8% to 67.7% depending on the parameter measured. Dynamic enzyme increases had poor accuracies, sensitivities, and NPVs overall, as shown in Table 3.

The test performance characteristics of decreases in dynamic liver enzyme in the 72 h preceding the procedure are summarized in Table 4, where both a minimum 30% decrease in enzymes and minimum 50% decrease were considered. Decreases in bilirubin or ALP of at least 50% were highly specific for the absence of CDL, with specificities of 91.7% (95% CI 85.7–95.8%) and 100.0% (97.2–100.0%), respectively. The combination of bilirubin and ALT both decreasing by at least 50% was also specific for the absence of CDL, with a specificity of 98.4% (95% CI 94.5–99.8%). Decreases in dynamic enzyme had low–moderate NPVs for ruling out CDL, ranging from 68.9% to 72.2%, and low-moderate accuracies for ruling out CDL, ranging from 51.1% to 71.6%.

**Figure 2:**
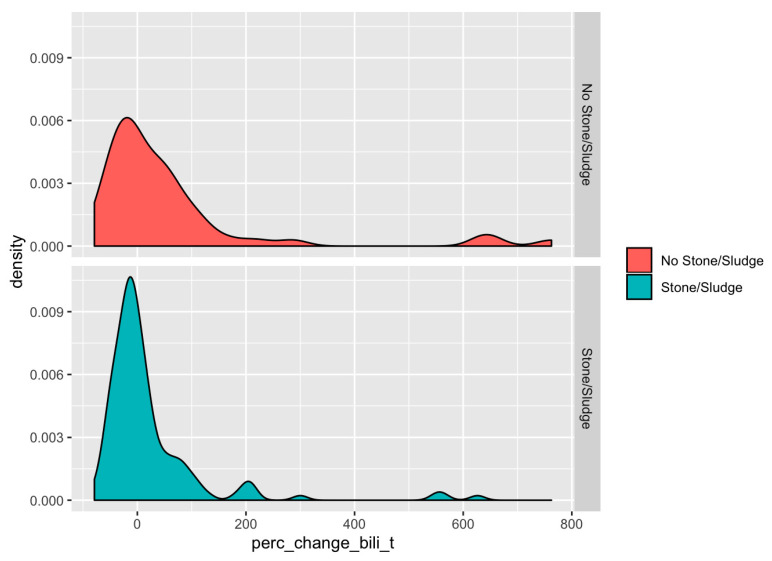
Density plots of percent change in total bilirubin for patients with and without an eventual finding of choledocholithiasis.

Density plots of the distribution of percent change of total bilirubin (Figure 2), ALT, and ALP (Supplementary Materials) demonstrated that, overall, there is no strong discriminatory power of biliary enzyme changes to discern between the presence or absence of CDL.

The performance characteristics of the high- and intermediate-probability criteria from the three guidelines of interest, along with the numbers of patients in each risk category who were found to have choledocholithiasis, are summarized in Table 5. PPV high-risk criteria were moderate overall, ranging between 74.0% and 85.2%, whereas specificity for high-risk criteria was lower, ranging between 39.7% and 66.1%. Sensitivity was poor for all intermediate- and high-risk criteria, ranging from 27.7–37.6% and 46.3–67.7%, respectively.

## 4. Discussion

In this study, we examined the performance of dynamic liver enzyme trends in predicting both the presence and absence of CDL. Several liver enzyme trends appear to be specific to the absence or presence of stones; in particular, significant decreases in total bilirubin, ALP, or both bilirubin and ALT of at least 30–50% within the prior 72 h appear to be especially predictive of the absence of intraductal findings during endoscopy. However, overall, the positive and negative predictive values of dynamic enzyme trends are low–moderate. Furthermore, using our in-patient cohort, we assessed the performance characteristics of several guideline-based CDL risk prediction criteria, with our findings confirming only modest predictive capacities, as previously demonstrated. Taken together, our findings would suggest that there may yet be a role for the incorporation of dynamic liver enzyme trends into future CDL guidelines and risk prediction models, but that these trends should not replace current guideline-based recommendations in the clinical pathway of patients with suspected CDL.

Several previous studies have examined single time point liver enzymes as a predictor of CDL; as a result, these markers (bilirubin in particular) have long been employed in ASGE CDL criteria as high-risk predictors [3,6]. Similarly, the 2019 ESGE criteria include abnormal liver enzymes as an intermediate-risk predictor [7]. Far fewer studies have examined the role of dynamic liver enzyme trends as an adjunct to the existing prediction rules. In 2015, Adams et al. published a study assessing the performance of measuring two sets of liver enzymes prior to their confirmatory examination [14]. After applying the 2010 ASGE criteria, the majority (77.2%) of their initially high-risk patients maintained this designation, while 22.8% were downgraded to either the intermediate- or low-risk category upon consideration of the second set of enzymes. Conversely, 10% of the patients who were initially designated as intermediate- or low-risk were reclassified as high-risk after the second set. Of note, they demonstrated that a decrease in both bilirubin and ALT of 30% between the two sets of liver enzymes predicted a spontaneously passed stone with an overall accuracy of 45.3%. However, this study was limited by significant variations in the timing between repeat measurement of liver enzymes, with a significant proportion of patients having enzymes measured only 6 h apart [14]. In a similar fashion, Suarez et al. published a 2016 study assessing the value of measuring a second set of liver enzymes [20]. In their cohort, 64.8% of the initially high-risk patients maintained their classification, and 12.8% of the previously intermediate- or low-risk patients were reclassified as high-risk. They also demonstrated that a decrease in both bilirubin and ALT of 30% predicted a spontaneously passed stone with a 45.2% accuracy, and reported that a second set of enzymes did not improve accuracy, which was 62.7% [20]. In 2018, Panda et al. published a study assessing liver enzyme trends in the prediction of retained CBD stones in patients with acute gallstone pancreatitis. [17] However, this study was limited by a small sample size, modeled only the prediction of the presence of retained stones (and not the absence of stones), and was less generalizable overall given it was conducted among only gallstone pancreatitis patients [17]. In a small 2019 study of fewer than 60 patients, Gillaspie et al. did not conclude any predictive utility of trends in total bilirubin [18]. Finally, in a 2019 prospective cohort study published by Yu et al., neither a 20% change in liver enzymes nor an alternating pattern of liver enzyme changes (hypothesized to be reflective of a ball-valve effect of the stone) within the first three sets of liver enzymes measured were reliably predictive of CDL [19].

Analysis of our relatively homogeneous in-patient cohort demonstrates that decreases in ALP, bilirubin, or ALT of 50% or more within 72 h of endoscopy predict the absence of CDL with high specificity, a novel finding. Acknowledging the ball-valve theory of impacted CDL, this finding could suggest the feasibility of less-urgent out-patient stratification imaging (EUS or MRCP) for intermediate-probability patients in centers where urgent in-patient EUS or MRCP is a limited resource, and thus patients may otherwise proceed to ERCP without stratification. Similarly, but somewhat more modestly, increases in liver enzymes of at least 50% were predictive of the presence of CDL. Important differences in methodology likely explain why our findings were positive, where some others have not demonstrated the effectiveness of measuring liver enzyme trends. Importantly, we indexed the measurement of liver enzymes from the day of the ultimate endoscopic procedure, rather than from the day of admission, thus allowing us to set a strict 72 h window leading up to procedures during which trends could be measured, ideally as far apart within this window as possible. Secondly, we set more stringent cut-offs of 30% and 50% in our definitions of significant enzyme changes. Thirdly, we carefully excluded out-patients, patients with missing endoscopic data, or those without clear procedural indications suggesting possible CBD stones. In so doing, we performed our analyses on a fairly homogeneous cohort that is still generalizable to any settings in which the utility of dynamic liver enzyme measurement would potentially be useful.

In our assessment of society-based criteria, we found similar modest performance characteristics of existing guidelines compared with those reported in prior validation studies, including those assessing the more contemporary 2019 ASGE guidelines [10,15,21]. While our assessment of existing criteria using our cohort is not novel, it underscores the need for more robust predictors for CDL, especially for patients at intermediate risk. Thus, the strengths of our study include its homogenous but still generalizable dataset, our novel finding of the specificity of some dynamic enzyme trends when previous studies have not shown this, and our confirmation of the modest predictive utility of the current existing guidelines on a real-world dataset.

Despite the several strengths of our study design, there are also several limitations that should be acknowledged. Firstly, the study is retrospective, and thus a significant degree of bias is likely to be present at baseline. Specifically, the overall clinical ‘gestalt’ at the time of evaluation in certain patients may have dictated the request for enzyme trending, which could have imposed significant bias, favoring trending in patients at higher risk (e.g., an enzyme trend may have been ordered for patients still suffering from abdominal pain the day after admission, while those with ball-valving stones and intermittent decompression of the biliary tree may have been falsely thought to have passed a stone). Secondly, despite the high volume of our center, we were only able to provide a relatively low sample size for those with complete data points within 72 h of presentation. Similarly, given the retrospective design, patients with missing or incomplete data were also excluded, further limiting the sample size. Thirdly, era and operator effects are also likely present, given that our inpatient ERCP coverage is shared between providers familiar and unfamiliar with EUS. This may have swayed the request for follow-up liver enzymes when an ERCP-only trained provider was staffing the in-patient procedural requests. Similarly, between 2012 and 2018, practice patterns could have conceivably changed between the start and end of our timeframe. We plan on assessing the test performance characteristics of dynamic enzyme changes prospectively using prospective data from 2018 onward [16], at which time potential temporal changes in practice will also be assessed. Fourthly, we opted to include cases in which either sludge and/or stones were found in the biliary tree, but one must recognize that dynamic enzyme trends could perform differently if only stones were considered positive findings. Finally, though we employed strict eligibility criteria to create as homogeneous a study population as possible, these strict criteria could potentially limit the generalizability (external validity) of our findings when applied to other patient populations such as out-patients or those with prior sphincterotomies.

Although future research is warranted prior to formally integrating dynamic enzyme trends into guideline-based criteria, clinicians can nevertheless consider our findings to help triage the use of in-patient resources. For example, patients deemed at intermediate risk for the presence of CDL but in whom dynamic enzyme trends suggest their absence could potentially be scheduled for less invasive and/or less urgent follow-up approaches. This approach could serve to streamline in-patient resources and control healthcare expenditure while improving patient safety outcomes. In conclusion, dynamic liver enzyme trends may play an important role in optimizing the risk stratification of patients at risk of CDL, though future prospective studies are needed to better delineate their performance, reliability, and cost-effectiveness in routine clinical practice.

## Figures and Tables

**Table 1:** Summary of ASGE 2010, ASGE 2019, and ESGE 2019 recommendations regarding the pre-test probability of choledocholithiasis.

Probability	ASGE 2010 [3]	ASGE 2019 [6]	ESGE 2019 [7]
High (>50%)	(1) CBD stone on US and/or (2) Clinical ascending cholangitis and/or (3) Bilirubin > 4 mg/dL and/or (4) Bilirubin 1.8–4 mg/dL AND CBD > 6 mm on US	(1) CBD stone on US or cross-sectional imaging and/or (2) Clinical ascending cholangitis and/or (3) Bilirubin > 4 mg/dL AND CBD > 6 mm on US	(1) Clinical ascending cholangitis and/or (2) CBD stone on US
Intermediate (10–50%)	(1) Bilirubin 1.8–4 mg/dL and/or (2) CBD > 6 mm on US and/or (3) Abnormal liver biochemical tests other than bilirubin and/or (4) Clinical gallstone pancreatitis and/or (5) Age > 55 years	(1) Bilirubin ≥ 1.8 mg/dL and/or (2) CBD > 6 mm on US and/or (3) Abnormal liver biochemical tests other than bilirubin and/or (4) Clinical gallstone pancreatitis and/or (5) Age > 55 years	(1) Any abnormal liver biochemical tests and/or (2) CBD > 6 mm on US
Low (<10%)	None of the above predictors present	None of the above predictors present	None of the above predictors present

ASGE, American Society for Gastrointestinal Endoscopy; ESGE, European society for gastrointestinal endoscopy; CBD, common bile duct; US, ultrasound.

**Table 2:** Baseline patient, procedure, biochemical, and imaging parameters of the 879 patients analyzed as part of the study cohort.

	Patients with Positive Diagnosis of Choledocholithiasis (*n* = 622)	Patients with Negative Diagnosis of Choledocholithiasis (*n* = 257)	*p*-Value
Sex			0.20
Female % (*n*)	60.0 (373)	55.3 (142)	
Male % (*n*)	40.0 (249)	44.7 (115)	
Mean age (SD)	59.3 (21.2)	66.0 (18.2)	<0.001
Procedure performed			<0.001
ERCP % (*n*)	97.7 (608)	86.0 (221)	
EUS % (*n*)	2.3 (14)	14.0 (36)	
Pre-Procedure Imaging			
Yes % (*n*)	78.9 (472)	82.9 (213)	
No % (*n*)	24.1 (150)	17.1 (44)	
Stone seen on imaging			<0.001
Yes % (*n*)	54.7 (340)	0.0 (0)	
No % (*n*)	45.3 (282)	100.0 (257)	
CBD > 6 mm			0.04
Yes % (*n*)	55.3 (344)	47.9 (123)	
No % (*n*)	44.7 (278)	52.1 (134)	
Mean CBD size in mm, of those >6 mm (SD)	12.4 (4.5)	11.6 (3.8)	0.01
Mean total bilirubin in µmol/L (SD)	64.9 (62.1)	60.6 (63.3)	0.35
Mean ALT in U/L (SD)	292.4 (346.6)	283.6 (267.0)	0.72
Mean ALP in U/L (SD)	278.2 (256.5)	261.1 (191.4)	0.75
2010 ASGE guideline risk category			0.01
High % (*n*)	67.7 (421)	57.6 (148)	
Intermediate % (*n*)	27.7 (172)	33.5 (86)	
Low % (*n*)	4.7 (29)	8.9 (23)	

SD, standard deviation; ERCP, endoscopic retrograde cholangiopancreatography; EUS, endoscopic ultrasound; CBD, common bile duct; ALT, alanine transferase; ALP, alkaline phosphatase; ASGE, American Society for Gastrointestinal Endoscopy.

**Table 3:** Performance characteristics of an *increase* in liver enzymes within 72 h preceding ERCP or EUS as a predictor of *the presence of choledocholithiasis*.

	Accuracy % (95% CI)	Sensitivity % (95% CI)	Specificity % (95% CI)	PPV % (95% CI)	NPV % (95% CI)	Patients Used in Models
Bilirubin **increase** **of at least 30%**	33.5	24.1	57.7	59.3	22.9	
	(26.8–40.8)	(17.1–32.2)	(43.2–71.3)	(45.0–72.4)	(16.0–31.1)	190
Bilirubin **increase** **of at least 50%**	31.9	19.5	63.5	57.8	23.6	
	(25.2–39.1)	(13.2–27.3)	(49.0–76.4)	(42.2–72.3)	(16.8–31.5)	190
ALP **increase** **of at least 30%**	32.2	12.2	82.7	64.0	27.2	
	(25.5–39.5)	(7.1–19.1)	(69.7–91.8)	(42.5–82.0)	(20.4–34.9)	187
ALP **increase** **of at least 50%**	29.5	6.1	88.5	57.1	27.2	
	(23.0–36.7)	(2.7–11.7)	(76.6–95.6)	(28.9–82.3)	(20.7–34.6)	187
ALT **increase** **of at least 30%**	34.6	19.8	72.5	65.0	26.1	
	(27.7–42.0)	(13.4–27.7)	(58.3–84.1)	(48.3–79.4)	(19.1–34.1)	186
ALT **increase** **of at least 50%**	34.1	16.0	80.4	67.7	27.2	
	(27.2–41.4)	(10.2–23.5)	(66.9–90.2)	(48.6–83.3)	(20.2–35.0)	186
Bilirubin OR ALT **increase of at least 30%**	37.4	32.0	51.0	62.1	23.0	
	(30.3–45.0)	(24.1–40.9)	(36.6–65.2)	(49.3–73.8)	(15.6–31.9)	183
Bilirubin OR ALT **increase of at least 50%**	34.6	25.0	58.8	60.4	23.8	
	(27.7–42.1)	(17.8–33.4)	(44.2–72.4)	(46.0–73.5)	(16.7–32.2)	183
Bilirubin AND ALT **increase of at least 30%**	31.3	12.5	78.4	59.3	26.3	
	(24.6–38.6)	(7.3–19.5)	(64.7–88.7)	(38.8–77.6)	(19.5–34.1)	183
Bilirubin AND ALT **increase of at least 50%**	31.8	10.9	84.3	63.6	27.4	
	(25.1–39.2)	(6.1–17.7)	(71.4–93.0)	(40.7–82.8)	(20.6–35.1)	183
Any enzyme **increase** **of at least 30%**	37.9	34.7	46.0	61.4	22.1	
	(30.7–45.6)	(26.4–43.7)	(31.8–60.7)	(49.0–72.8)	(14.6–31.3)	177
Any enzyme **increase** **of at least 50%**	35.1	25.8	80.4	60.4	24.0	
	(28.0–42.6)	(18.4–34.4)	(66.9–90.2)	(46.0–73.5)	(16.7–32.6)	177

CI, confidence interval; PPV, positive predictive value; NPV, negative predictive value; ALP, alkaline phosphatase; SD, standard deviation; ERCP, endoscopic retrograde cholangiopancreatography; EUS, endoscopic ultrasound; ALT, alanine transferase. Point estimates over 80% highlighted.

**Table 4:** Performance characteristics of a *decrease* in liver enzymes within 72 h preceding ERCP or EUS as a predictor of *the absence of choledocholithiasis*.

	Accuracy % (95% CI)	Sensitivity % (95% CI)	Specificity % (95% CI)	PPV % (95% CI)	NPV % (95% CI)	Patients Used in Models
Bilirubin **decrease** **of at least 30%**	61.6	21.2	77.4	26.8	71.5	
	(54.2–68.7)	(11.1–34.7)	(69.4–84.2)	(14.2–42.9)	(63.4–78.7)	190
Bilirubin **decrease** **of at least 50%**	68.6	9.6	91.7	31.2	72.2	
	(61.4–75.3)	(3.2–21.0)	(85.7–95.8)	(11.0–58.7)	(64.8–78.8)	190
ALP **decrease** **of at least 30%**	67.2	1.9	93.1	10.0	70.5	
	(59.9–74.0)	(0.0–10.3)	(87.4–96.8)	(0.3–44.5)	(63.1–77.2)	187
ALP **decrease** **of at least 50%**	71.6	0.0	100.0	N/R	71.6	
	(64.5–78.0)	(0.0–6.8)	(97.2–100.0)		(64.5–78.0)	187
ALT **decrease** **of at least 30%**	55.5	19.6	69.5	20.0	68.9	
	(48.0–62.8)	(9.8–33.1)	(60.8–77.2)	(10.0–33.7)	(60.3–76.7)	186
ALT **decrease** **of at least 50%**	64.8	3.9	88.5	11.8	70.3	
	(57.4–71.8)	(0.5–13.5)	(81.8- 93.4)	(1.5–36.4)	(62.7–77.2)	186
Bilirubin OR ALT **decrease of at least 30%**	52.0	35.3	58.6	25.4	69.4	
	(44.4–59.5)	(22.4–49.9)	(49.6–67.2)	(15.8–37.1)	(59.8–77.9)	183
Bilirubin OR ALT **decrease of at least 50%**	62.0	11.8	82.0	20.7	69.7	
	(54.5–69.1)	(4.4–23.9)	(74.3–88.3)	(8.0–39.7)	(61.5–77.0)	183
Bilirubin AND ALT **decrease of at least 30%**	64.8	3.9	89.1	12.5	69.9	
	(57.3–71.8)	(0.5–13.5)	(82.3–93.9)	(1.6–38.3)	(62.3–76.9)	183
Bilirubin AND ALT **decrease of at least 50%**	70.4	0.0	98.4	0.0	71.2	
	(63.1–77.0)	(0.0–7.0)	(94.5–99.8)	(0.0–8.4)	(63.9–77.7)	183
Any enzyme **decrease** **of at least 30%**	51.1	36.0	57.3	25.4	68.9	
	(43.5–58.8)	(22.9–50.8)	(48.1–66.1)	(15.8–37.1)	(59.1–77.7)	177
Any enzyme **decrease** **of at least 50%**	61.5	12.0	81.5	20.7	69.7	
	(53.8–68.8)	(4.5–24.3)	(73.5–87.9)	(8.0–39.7)	(61.5–77.0)	177

CI, confidence interval; PPV, positive predictive value; NPV, negative predictive value; ALP, alkaline phosphatase; SD, standard deviation; ERCP, endoscopic retrograde cholangiopancreatography; EUS, endoscopic ultrasound; ALT, alanine transferase. N/R, not reportable owing to a low positive event rate. Point estimates over 80% highlighted.

**Table 5:** Performance characteristics of endoscopy-society choledocholithiasis guidelines when applied to the study cohort.

	Patients with Choledocholithiasis/Total Patients in Risk Category	Sensitivity % (95% CI)	Specificity % (95% CI)	PPV % (95% CI)	NPV % (95% CI)
ASGE 2010—high risk [3]		67.7	57.6	74.0	35.2
	421/569	(64–71.4)	(36.4–48.5)	(70.4–77.6)	(29.8–40.5)
ASGE 2010—intermediate risk [3]		27.7	33.5	66.7	27.5
	172/258	(24.1–31.2)	(60.8–72.3)	(60.9–72.4)	(24–31.1)
ASGE 2019—high risk [6]		59.5	39.7	78.4	38.1
	370/472	(55.6–63.3)	(54.3–66.3)	(74.7–82.1)	(33.3–42.8)
ASGE 2019—intermediate risk [6]		35.9	54.1	61.6	22.8
	223/362	(32.1–39.6)	(39.8–52)	(56.6–66.6)	(19.2–26.4)
ESGE 2019—high risk [7]		46.3	19.5	85.2	38.3
	288/338	(42.4–50.2)	(75.7–85.4)	(81.4–89)	(34.2–42.4)
ESGE 2019—intermediate risk [7]		37.6	66.1	57.9	18.3
	243/404	(33.8–41.4)	(28.1–39.6)	(53.1–62.7)	(14.8–21.8)

CI, confidence interval; ASGE, American Society for Gastrointestinal Endoscopy; ESGE, European Society for Gastrointestinal Endoscopy; PPV, positive predictive value; NPV, negative predictive value. Point estimates over 80% highlighted.

## Data Availability

Data are potentially available upon reasonable request to the corresponding author.

## Supplementary Materials

The following supporting information can be downloaded at: https://www.mdpi.com/article/10.3390/jcm11154575/s1, Figure S1: Density plots of percent change of alkaline phosphatase for patients with and without an eventual finding of choledocholithiasis. Figure S2: Density plots of percent change of alanine transaminase for patients with and without an eventual finding of choledocholithiasis.

## Abbreviations

AE	adverse event
ALP	alkaline phosphatase
ALT	alanine transaminase
ASGE	American Society for Gastrointestinal Endoscopy
AST	aspartate transaminase
CBD	common bile duct
CDL	choledocholithiasis
CT	computed tomography
ERCP	endoscopic retrograde cholangiopancreatography
ESGE	European Society of Gastrointestinal Endoscopy
EUS	endoscopic ultrasound
GGT	gamma-glutamyltransferase
MRCP	magnetic resonance cholangiopancreatography
NPV	negative predictive value
PPV	positive predictive value
