# Supplementary material for: Test Performance Characteristics of Dynamic Liver Enzyme Trends in the Prediction of Choledocholithiasis
Source: J Clin Med. 2022 Aug 5;11(15):4575. doi: 10.3390/jcm11154575 (PMC9369577; doi:10.3390/jcm11154575)
Supplement: Supplementary file 1 [file jcm-11-04575-s001.zip › jcm-1753606-supplementary.pdf]

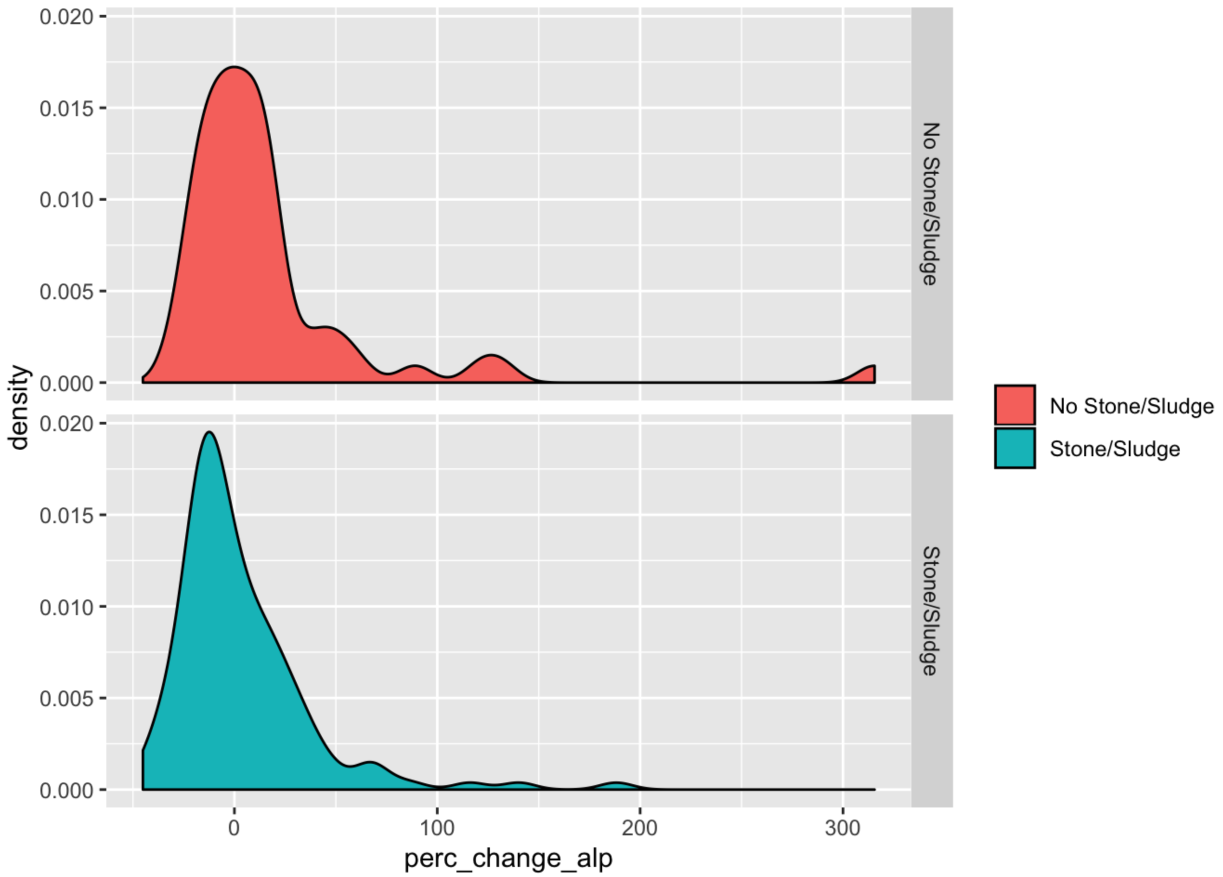

Figure S1. Density plots of percent change of alkaline phosphatase for patients with and without an eventual finding of choledocholithiasis.

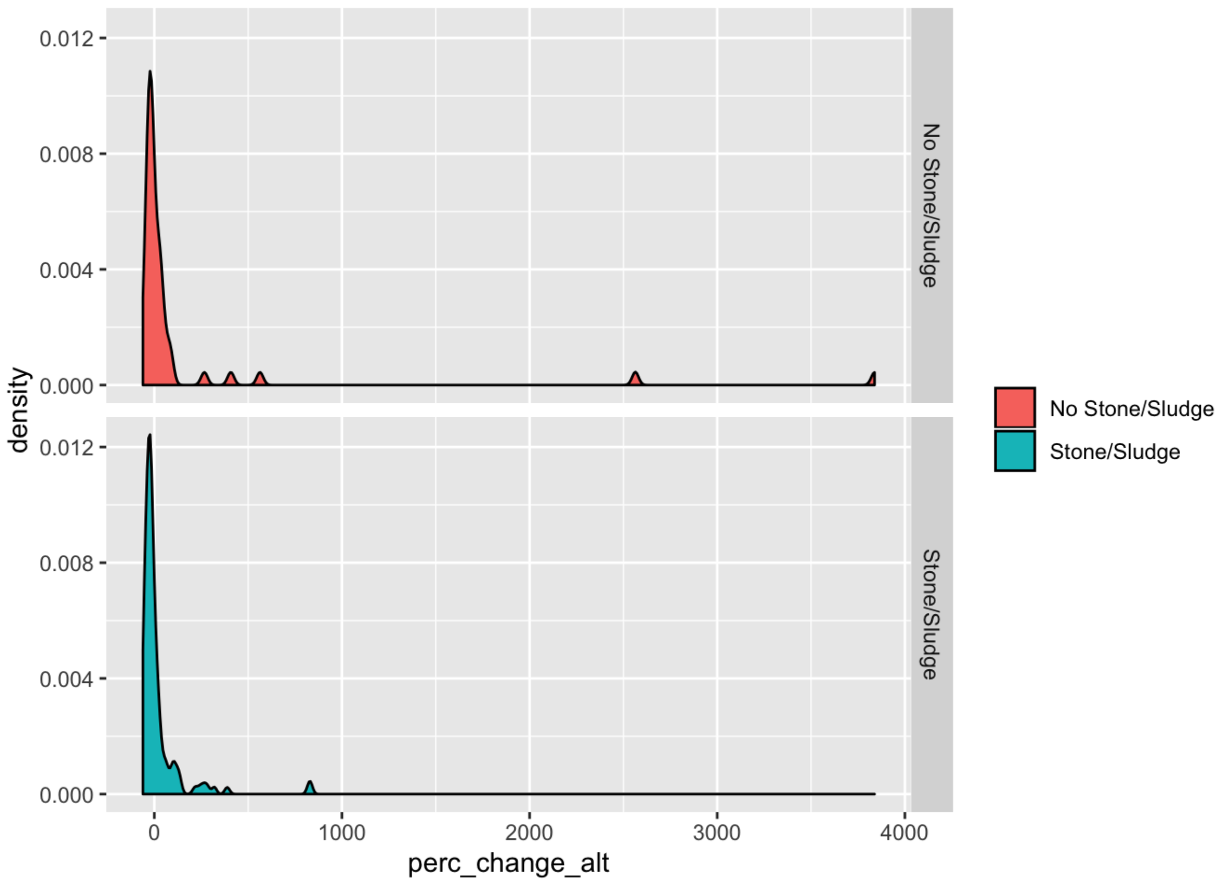

Figure S2. Density plots of percent change of alanine transaminase for patients with and without an eventual finding of choledocholithiasis.
